# Therapeutic Opportunities of Targeting Histone Deacetylase Isoforms to Eradicate Cancer Stem Cells

**DOI:** 10.3390/ijms19071939

**Published:** 2018-07-02

**Authors:** Peng-Chan Lin, Hao-Yu Hsieh, Po-Chen Chu, Ching S. Chen

**Affiliations:** 1 Department of Internal Medicine, National Cheng Kung University Hospital, College of Medicine, National Cheng Kung University, Tainan 70403, Taiwan; pengchanlin@gmail.com; 2 School of Pharmacy, College of Medicine, National Taiwan University, Taipei 10050, Taiwan; zarck104@hotmail.com; 3 Department of Cosmeceutics and Graduate Institute of Cosmeceutics, China Medical University, Taichung 40402, Taiwan; popopi@gmail.com; 4 Drug Development Center, China Medical University, Taichung 40402, Taiwan; 5 Department of Medical Research, China Medical University Hospital, China Medical University, Taichung 40447, Taiwan

**Keywords:** histone deacetylases, cancer stem cells, non-histone targets, acetylation status, chaperon proteins, transcription factors

## Abstract

Cancer stem cells (CSCs), or tumor-initiating cells, are a small subset of cancer cells with the capacity for self-renewal and differentiation, which have been shown to drive tumor initiation, progression, and metastasis in many types of cancer. Moreover, therapeutic regimens, such as cisplatin and radiation were reported to induce the enrichment of CSCs, thereby conferring chemoresistance on cancer cells. Therefore, therapeutic targeting of CSCs represents a clinical challenge that needs to be addressed to improve patient outcome. In this context, the effectiveness of pan or class-I histone deacetylase (HDAC) inhibitors in suppressing the CSC population is especially noteworthy in light of the new paradigm of combination therapy. Evidence suggests that this anti-CSC activity is associated with the ability of HDAC inhibitors to target multiple signaling pathways at different molecular levels. Beyond chromatin remodeling via histone acetylation, HDAC inhibitors can also block key signaling pathways pertinent to CSC maintenance. Especially noteworthy is the ability of different HDAC isoforms to regulate the protein stability and/or activity of a series of epithelial-mesenchymal transition (EMT)-inducing transcription factors, including HIF-1α, Stat3, Notch1, β-catenin, NF-κB, and c-Jun, each of which plays a critical role in regulating CSCs. From the translational perspective, these mechanistic links constitute a rationale to develop isoform-selective HDAC inhibitors as anti-CSC agents. Thus, this review aims to provide an overview on the roles of HDAC isoforms in maintaining CSC homeostasis via distinct signaling pathways independent of histone acetylation.

## 1. An Overview of Anti-Cancer Stem Cell (CSC) Strategies

**Figure 1:**
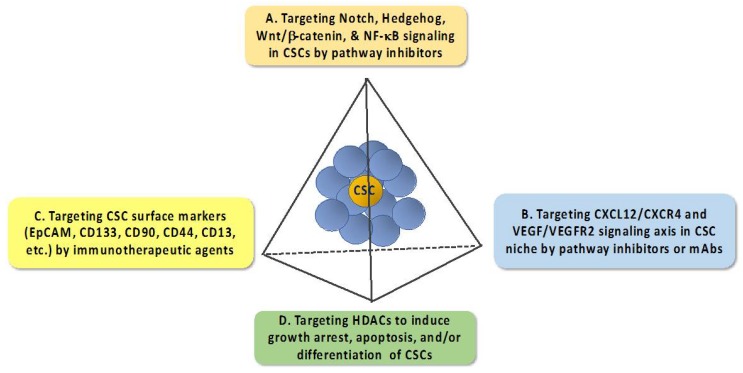
Diagrammatic scheme outlining major anti-cancer stem cell (CSC) strategies.

Cancer stem cells (CSCs), or tumor-initiating cells, represent a small subset of undifferentiated tumor cells characterized by their tumorigenic properties and capacity for self-renewal and differentiation [1,2]. The concept of CSCs has provided a new paradigm to understand the cellular process that drives tumor initiation, progression, metastasis, and therapy resistance in many types of cancer. For example, CSCs have adopted multiple self-defense mechanisms to develop intrinsic chemo/radio-resistant phenotypes, including CSC niche, increases in the expression of ATP-binding cassette transporters to increase drug efflux, increases in the expression of drug-inactivating enzymes, quiescence and dormancy, activation of DNA repair machinery, activation of pro-survival signaling pathways, and induction of epithelial–mesenchymal transition (EMT) through microRNAs, and plasticity [3,4]. In addition, recent reports indicate that the CSC subpopulation could be enriched in response to cytotoxic agents or radiation treatment [5,6]. Mechanistically, cisplatin was reported to enhance CSCs by upregulating the expression of the oncogene TRIB1 that might be involved in regulating CSC maintenance and multidrug resistance [5], and radiotherapy might increase CSCs by facilitating the dedifferentiation of non-stem cancer cells into CSCs via EMT [6]. Consequently, as CSCs are more resistant to chemotherapeutic agents than the non-CSC population within a tumor, this enrichment enables the surviving CSCs to repopulate the tumor, leading to cancer relapse. Therefore, how to eradicate the CSC subpopulation represents an unmet medical need that warrants attention to improve clinical outcomes. To date, a number of strategies have been developed to suppress CSCs via different strategies (Figure 1) [7,8,9], which are briefly described as follows. 

### 1.1. Targeting Pathways that Regulate the Expression of EMT—Inducing Transcription Factors

The past decade has witnessed rapid advances in the understanding of the complex network of signaling pathways that govern the tumorigenic properties and the self-renewal capacity of CSCs. Especially, the intricate link between EMT and CSCs in driving tumor heterogeneity is noteworthy as EMT promotes the ability of cancer cells to acquire CSC properties [10,11]. Consequently, many signaling pathways that regulate the expression of EMT-inducing transcription factors, such as transforming growth factor (TGF)-β, Snail/Slug, Twist, and Zeb1/2, are functionally linked to the maintenance of CSC populations [12,13,14,15,16]. These pathways include those mediated by Notch, Hedgehog, Wnt/β-catenin, and NF-κB [17]. Mechanistically, targeting these signaling pathways might represent a viable strategy for CSC elimination (Figure 1), which is manifested by the clinical trials of many of these pathway inhibitors in different types of cancer [8]. 

### 1.2. Targeting the CXCL12–CXCR4 Signaling Axis

Substantial evidence has demonstrated the involvement of the tumor microenvironment in facilitating CSC growth, metastasis, and chemoresistance through the CXCL12–CXCR4 signaling axis via an autocrine- or paracrine-dependent mechanism [18]. Moreover, evidence indicates that CXCL12 [also known as stromal-derived factor-1 (SDF-1)] can also promote angiogenesis by stimulating CXCR4-positive cancer cells to secrete VEGF and IL-6 [19], and that this chemokine contributes to the ability of tumor cells to evade immune surveillance by regulating the trafficking of immune cells [20]. Thus, the CXCL12–CXCR4 signaling axis has been the focus of many drug discovery efforts, which has netted a series of CXCL12- or CXCR4-antagonizing small-molecule agents, aptamers, or peptides under preclinical development.

### 1.3. Targeting CSC Surface Markers

A number of cell surface markers are differentially expressed between CSCs and normal cells. Therapeutically, these CSC surface markers could be exploited to develop anti-CSC immunotherapeutic agents [21,22,23]. To date, a plethora of CSC surface markers have been identified in different types of CSCs, including, but not limited to, EpCAM, CD133, CD90, CD44, and CD13 [9,24]. Some of these CSC cell surface makers have been used as cancer vaccines, and monoclonal antibodies against these surface markers could be used as neutralizing antibodies or to prepare cytotoxic drug conjugates for CSC-targeted therapy. Moreover, short peptides that bound CSC surface markers were identified via the phage display technology, including those targeting CD133 [25] and CD44 [26]. In principle, these short peptides could be loaded onto nanoparticles/liposomes for CSC-targeted delivery of cytotoxic agents.

### 1.4. Targeting Histone Deacetylases

Among various anti-CSC strategies, the ability of histone deacetylase (HDAC) inhibitors, alone or in combination therapy, to decrease tumor aggressiveness by eradicating CSCs is intriguing [27,28,29]. To date, a large number of pan or class I HDAC inhibitors have been developed, many of which are undergoing different stages of clinical trials or are approved for clinical use (readers are referred to recent reviews for a list of HDAC inhibitors, their classifications, and clinical statuses [30,31,32]). A number of broad-spectrum HDAC inhibitors have been reported to suppress the CSC population in different cancer cell lines via distinct mechanisms. For example, AR-42 (OSU-HDAC42) was effective in causing apoptosis in leukemic stem cells, but not normal hematopoietic stem and progenitor cells, through the concomitant inhibition of NF-κB and Hsp90 functions [33]. SAHA could reduce the self-renewal capacity of pancreatic CSCs, in part through the inhibition of miR-34a-Notch signaling and EMT [34]. SAHA was also shown to reverse cisplatin resistance in head and neck cancer cells, which was linked to its ability to decrease CSCs via the downregulation of Nanog expression [35]. Moreover, abexinostat, another pan-HDAC inhibitor, was reported to reduce the CSC population through the induction of differentiation in breast cancer cell lines exhibiting low abundance of the long noncoding RNA Xist [36]. More recently, the newly developed pan-HDAC inhibitors MC1742 and MC2625 were shown to be effective in inducing growth arrest, apoptosis, and differentiation in sarcoma CSCs [37]. When combined with the DNA methyltransferase (DNMT) inhibitor 5-azacytidine, sodium butyrate was highly effective in reducing CSC abundance in breast tumors, in part by blocking the expression of growth-promoting signaling molecules, such as RAD51AP1 and SPC25 [38]. 

**Figure 2:**
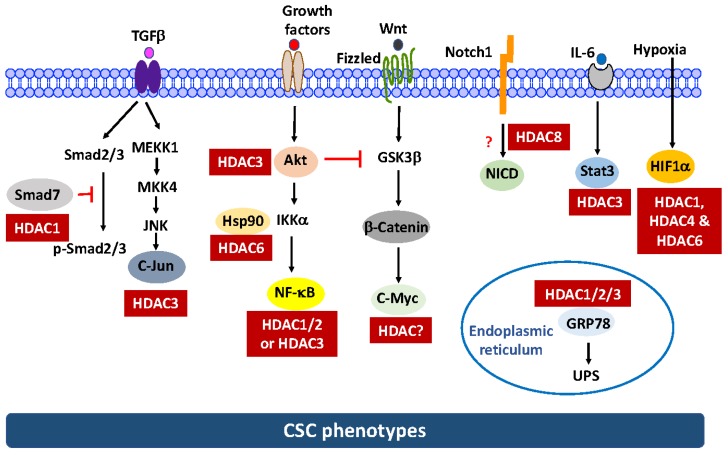
Role of histone deacetylase (HDAC) isoforms in regulating signaling effectors pertinent to CSC maintenance. The mechanism by which HDAC8 regulates the stability of Notch1 and the identity of the HDAC isoform responsible for c-Myc deacetylation remain undefined and are thus represented by dashed lines. The red question marks and red T-bar arrows denote unknown mechanism/isoform identity and negative regulation, respectively.

Despite these advances, two issues warrant attention with respect to the anti-CSC activities of these HDAC inhibitors. First, the mechanism by which these HDAC inhibitors suppress the CSC population has not been fully elucidated due to the complexity of the antitumor mechanism of HDAC inhibitors [30]. Mechanistically, the antitumor activity of HDAC inhibitors is attributable to their epigenetic effect on the reprogramming of gene expression in cancer cells, which leads to growth arrest, differentiation, and apoptosis [39]. However, how these changes affect the CSC population remains to be elucidated. One school of thought is that HDAC inhibitors could suppress the self-renewal capability and drive the differentiation of CSCs, thereby enhancing their sensitivity to chemo/radiotherapy [29]. Recent evidence suggests that non-CSCs could be induced into drug-resistant CSCs in response to chemotherapy and that this drug-induced CSC plasticity might be associated with the upregulation of HDAC expression [40]. Consequently, pharmacological inhibition of HDACs might disrupt CSC plasticity and restore drug sensitivity [40]. In addition, substantial evidence indicates that HDACs could facilitate the deacetylation of an array of non-histone targets in various signaling pathways relevant to CSC homeostasis, which might be cancer type-specific (Figure 2).

Second, there is a total of 11 Zn^2+^-dependent isoforms, which are classified into four groups (class I, HDAC1, 2, 3, 8; class IIa, HDAC4, 5, 7, 9; class IIb, HDAC6, 10; and class IV, HDAC11), each of which exhibits a distinct biological function in different cell types [39]. It remains unclear which of these 11 isoforms are responsible for the suppressive effect of pan-HDAC inhibitors on CSCs. Thus, this review summarizes data reported by this and other laboratories on the role of Zn^2+^-dependent HDAC isoforms in maintaining CSCs, which provides a mechanistic rationale for the development of HDAC isoform- or class-specific inhibitors in anti-CSC therapy. This article, however, does not discuss the role of the sirtuin class of HDACs (class III), which are NAD^+^-dependent protein deacetylases, in CSC regulation, as this topic has been addressed in recent reviews [41,42]. Also, the structures, classification, biological functions, and modes of mechanism of histone deacetylase isoforms have been extensively covered [39,43,44], and they will not be discussed here.

## 2. Multifaceted Molecular Mechanisms by Which HDAC Inhibitors Eradicate CSCs Independent of Histone Modifications

Reminiscent to their antitumor mechanisms, the anti-CSC activity of HDAC inhibitors is also attributable to a complex network of histone acetylation-dependent and -independent pathways, which involves the inhibition of multiple HDAC isoforms [30]. Beyond chromatin remodeling via histone acetylation, HDACs play a critical role in regulating various signaling pathways pertinent to CSC maintenance. Specifically, HDACs, especially class I HDACs (HDAC1-3, 8), are able to regulate the stability and/or activity of a host of chaperon proteins and transcription factors by controlling their acetylation status or through physical interactions (Figure 2). Alternatively, HDACs might also regulate the stability of key CSC regulators, such as β-catenin and Notch, by targeting their upstream effectors. As these non-histone targets are involved in regulating CSC homeostasis, interference of their functions underlies the anti-CSC activity of HDAC inhibitors. However, it should be noted that as these critical CSC-regulatory pathways are also shared by normal stem cells [4], the effects of HDAC inhibitors on these signaling pathways in the normal stem cell population remains to be interrogated.

To shed light onto the intricate roles of HDACs in promoting CSC phenotypes, interplays between HDAC isoforms with various non-histone targets might be mediated through two mechanisms, which are (A) direct acetylation and (B) targeting the acetylation of upstream effectors, as discussed below.

### 2.1. Regulation of the Protein Stability and/or Activity of Target Proteins Via Direct Acetylation

**(1) Hypoxia-inducible factor (HIF)-1α.** HIF-1α drives an array of cellular processes in tumor cells under hypoxic stress, including glycolytic switch, cell cycle progression, angiogenesis, and other aggressive behaviors, and its overexpression is correlated with poor prognosis in many types of cancer [45]. Recent evidence indicates that HIF-1α also plays a critical role in CSC regulation [46]. For example, HIF-1α was reported to upregulate Notch signaling by reversing a negative feedback regulation of the *Hes1* gene, a key Notch target involved in the self-renewal of CSCs [47]. In cancer cells, an intricate network of pathways has been reported to control the abundance and transcriptional activity of HIF-1α [48]. Under normoxic conditions, HIF1α is degraded via a hydroxylation/von Hippel-Lindau tumor suppressor (VHL)-dependent mechanism. Moreover, the protein stability and transcriptional activity of HIF-1α are also regulated by a protein acetylation–deacetylation system [49]. Specifically, ARD1 acetylates and reduces the protein stability of HIF1α [50], while several HDAC isoforms, including HDAC1 [51] and the class II isoforms HDAC4 and HDAC6 [52], were reported to act as HIF-1α deacetylase, which antagonize the effect of ARD1 on HIF-1α protein degradation. As a consequence, pharmacological inhibition or genetic knockdown of any of these HDAC isoforms resulted in the destabilization of HIF-1α.

**(2) Signal transducer and activator of transcription 3 (Stat3).** Evidence indicates that the IL-6/JAK/Stat3 pathway plays a critical role in the pathogenesis of breast cancer, and that dysregulated Stat3 activation promotes breast tumor progression due to overexpression of a plethora of target genes involved in cell survival, angiogenesis, and EMT [53]. Moreover, Stat3 is responsible for mediating the effect of IL-6 on CSC maintenance in human breast tumor cells [54]. Among various isoforms, HDAC3 was found to bind and deacetylate STAT3 [55]. Consequently, inhibition of HDAC3 abolished Stat3 phosphorylation at Try705 by increasing its acetylation at Lys685, leading to Stat3 inactivation [55].

**(3) c-Myc.** A recent report indicates that treatment of acute myeloid leukemia cells with HDAC inhibitors led to increased acetylation accompanied by the reduced protein stability of c-Myc [56]. However, it remains unclear which isoform was involved. As c-Myc plays a critical role in regulating the CSC population [57,58], identification of the HDAC isoform responsible for c-Myc deacetylation warrants investigations.

**(4) NF-κB.** NF-κB plays a critical role in CSC homeostasis due to the pivotal role of many of its target genes in regulating tumor initiation, recurrence, and metastasis [17]. Evidence indicates that multiple HDAC isoforms can regulate the transcriptional activity and/or stability of NF-κB through direct deacetylation or indirectly via the upstream kinases Akt and IκB kinase (IKK) α in the canonical pathway (Figure 2). Thus, inhibition of HDACs results in decreased NF-κB-mediated transcription. With respect to direct regulation, several HDAC isoforms have been reported to deacetylate the RelA subunit of NF-κB in different cell systems. For example, HDAC1/2 are involved in RelA deacetylation in Schwann cells [59], while HDAC3 acts as RelA deacetylase in HEK293 and HeLa cells [60,61,62]. However, it remains to be confirmed which isoform is responsible for RelA acetylation in CSCs. Moreover, HDAC3 and HDAC6 could also indirectly take part in the regulation of the activation and nuclear localization of NF-κB through the deacetylation of Akt [63] and HSP90 [64], respectively, which also warrants attention.

**(5) c-Jun.** The role of c-Jun in regulating the CSC population was demonstrated by a recent study that c-Jun serves as an intermediary effector in c-Jun N-terminal kinase (JNK) signaling to promote stem cell phenotype in triple-negative breast cancer (TNBC) cells via the upregulation of Notch1 [65]. It is noteworthy that HDAC3 acts as a repressor of c-Jun by interacting with the ε-domain of c-Jun to suppress its transcriptional activity [66].

**(6) Smad7.** Smad7 negatively regulates TGF-β-mediated phosphorylation of Smad2/3, thereby effectively blocking the immunosuppressive functions of TGF-β [67]. A recent study shows that Smad7 was also involved in the maintenance of the epithelial phenotype in ovarian CSCs [68]. HDAC1 facilitates the deacetylation of Smad7, leading to decreased stability of Smad7 by enhancing its ubiquitination [69].

**(7) GRP78.** This chaperone protein was reported to increase the CD44^hi^/CD24^lo^ phenotype in head and neck CSCs [70]. Class I HDACs (HDAC1/2/3) colocalized with GRP78 in the endoplasmic reticulum and the inhibition of individual HDACs resulted in GRP78 acetylation and selective activation of the unfolded protein response (UPR) [71], which has been mechanistically linked to CSC maintenance [72].

### 2.2. Targeting Key Signaling Pathways Governing the CSC Population

In addition to the direct effect on the function of non-histone target proteins by altering their acetylation status, HDAC inhibitors can also block key self-renewal pathways pertinent to CSC maintenance, including those mediated by Notch1 and Wnt/β-catenin (Figure 2), which is delineated as follows:

1. **Notch1**. Data from this and other laboratories indicated that AR-42, SAHA, and the class I HDAC inhibitor depsipeptide were able to suppress the CSC population in TNBC [73] and/or pancreatic cancer cells [34], in part by suppressing Notch1 expression. We obtained evidence that this Notch1 suppression in TNBC was attributable to the ability of HDAC inhibitors to facilitate the proteasomal degradation of Notch1 [73]. Pursuant to this finding, we interrogated the mechanistic link between individual class I isoforms (HDAC1-3 and 8) and this drug-induced Notch1 degradation via genetic knockdown and ectopic expression, which revealed HDAC8 to be the primary mediator for Notch1 degradation [73]. Interestingly, co-immunoprecipitation analysis indicated that HDAC8 did not form complexes with Notch1 and HDAC inhibition had no effect on Notch1 acetylation, suggesting that Notch1 was not a substrate for HDAC8 [73]. However, the signaling mechanism by which HDAC8 regulates the stability of Notch1 remains to be elucidated.
2. **β-Catenin**. We recently reported that genetic knockdown or pharmacological inhibition of HDAC3 was effective in eliminating the CSC population in TNBC cells [74]. We obtained evidence that HDAC3 is mechanistically linked to CSC homeostasis by increasing β-catenin expression through the Akt/GSK3β pathway. This finding is consistent with the report that HDAC3 binds and deacetylates Akt at Lys20, which increases Akt phosphorylation [63]. Consequently, HDAC3 inhibition leads to β-catenin degradation via the inactivation of Akt signaling.

## 3. HDAC Isoforms as Anti-CSC Targets—Challenges and Opportunities

Based on the above discussions, it is conceivable that the anti-CSC effect of pan-HDAC inhibitors is likely attributable to multiple signaling pathways in a cancer type- and/or inhibitor-specific manner, which might involve more than one HDAC isoform. Because different types of tumor cells might exhibit differential expression profiles of HDAC isoforms, the relative contribution of the aforementioned signaling mechanisms might differ. For example, our previous studies have demonstrated the important role of HDAC3 and HDAC8 in regulating CSC homeostasis in TNBC as genetic silencing or pharmacological inhibition of either isoform was effective in eradicating TNBC CSCs in vitro and in vivo [73,74]. However, this strategy might not work for other cancer types. This premise is exemplified by a recent report that showed the necessity of HDAC1 and HDAC7, a class IIa isoform, in maintaining CSCs in ovarian and breast CSCs [75]. This study indicated that HDAC1 and HDAC7 were specifically overexpressed in the CSC population of breast and ovarian cancer cells, and that concomitant targeting of HDAC1 and HDAC7 by MS-275 (Entinostat) and MGCD0103 (Mocetinostat) could effectively eliminate CSCs, as these class I-specific inhibitors could suppress HDAC7 expression by facilitating its degradation [75]. Together, these findings suggest that it might be more advantageous to use inhibitors with a broader spectrum of isoform selectivity (such as HDAC1, 3, and 8), in lieu of targeting a single isoform, as anti-CSC agents to overcome the cancer type-specificity issue.

In addition, other two issues concerning class II HDACs (HDAC4, 5, 7, 9) warrant clarification. First, relative to class I HDACs, the functional roles of class IIa isoforms in regulating the CSC phenotype remain elusive, which need to be addressed, as many of these class IIa enzymes were highly expressed and associated with poor prognosis in certain types of tumors [76,77,78]. In addition to the aforementioned HDAC7 in ovarian and breast CSCs [75], HDAC5 and HDAC9 have also been implicated in the maintenance of lung CSCs [79] and the pathogenesis of lymphoma in mice [80], respectively. However, relative to class I enzymes, we have little understanding of the biology of class IIa HDACs. Second, the interplay between different HDAC isoforms to regulate CSCs is intriguing. For example, the mechanism by which HDAC1 and HDAC7 act concertedly to maintain the stemness of ovarian and breast CSCs and the ability of class I inhibitors to downregulate the expression of HDAC7 warrant further investigation.

## 4. Conclusions

In this review article, we have discussed the critical role of HDAC isoforms, especially those of class I (HDAC1-3, 8), in maintaining CSC homeostasis via distinct signaling pathways independent of histone acetylation (Figure 2). Specifically, these HDAC isoforms regulate the protein stability and/or activity of a series of EMT-inducing transcription factors, including HIF-1α, Stat3, Notch1, β-catenin, NF-κB, and c-Jun, each of which plays a critical role in regulating CSCs. Among various class I HDACs, HDAC3 is noteworthy because it could simultaneously regulate multiple targets (Stat3, β-catenin, NF-κB, and c-Jun). From the translational perspective, these mechanistic links provide a rationale to develop isoform-selective HDAC inhibitors as anti-CSC agents. However, like many other targeted therapies, there are limitations associated with the therapeutic targeting of HDAC isoforms, in part, due to heterogeneity in CSC populations, which may rely upon different CSC-related genes for survival. In addition, an area that needs additional research is the effects of chemotherapeutic agents or radiation, which are known to enrich CSCs, on the HDAC isoform profiling in the CSC population. These changes may also contribute to the development of a therapy-resistant phenotype. Nevertheless, targeting HDAC isoforms represents a promising strategy for anti-CSC therapy, which warrants further investigation.
